# Does the Menstrual Phase Matter in Vascular Endothelial Responses to Acute Exercise? A Narrative Review of the Literature

**DOI:** 10.3390/sports13070210

**Published:** 2025-06-27

**Authors:** Sairos Ghniem, Ellen A. Dawson, Andrea Tryfonos

**Affiliations:** 1 Department of Life Sciences, School of Sciences, European University Cyprus, Nicosia 1516, Cyprus; s.ghniem1@gmail.com; 2 Research Institute for Sport and Exercise Science, Liverpool John Moores University, Liverpool L3 3AF, UK; e.dawson@ljmu.ac.uk; 3 Liverpool Center for Cardiovascular Science, Liverpool Centre for Cardiovascular Science at University of Liverpool, Liverpool John Moores University and Liverpool Heart & Chest Hospital, Liverpool L14 3PE, UK; 4 Division of Clinical Physiology, Department of Laboratory Medicine, Karolinska Institutet, 171 77 Stockholm, Sweden

**Keywords:** vascular responses, endothelial function, flow-mediated dilation, estrogen, menstrual cycle, acute exercise

## Abstract

Women have a lower age-matched cardiovascular risk than men, largely due to estrogen’s protective role in endothelial function. While exercise improves vascular health, acute vascular responses are influenced by factors such as age, fitness level, metabolic status, and exercise modality. In premenopausal women, fluctuations in estrogen levels during the menstrual cycle may further affect vascular reactivity. Here, we review current evidence on acute exercise-induced vascular responses in women, emphasizing menstrual phase influences and key biomarkers such as flow-mediated dilation (FMD), along with others including vascular conductance and pulse wave velocity (PWV). Despite limited and heterogeneous evidence, shear-induced vascular responses, (including FMD) following acute exercise, appear to be relatively stable across menstrual cycle phase, suggesting that strict phasic control may not always be necessary. However, future high-quality studies are needed to further clarify this response. In contrast, other vascular assessments that rely more heavily on neural components—such as vascular conductance and PWV—show greater estrogen sensitivity. Nonetheless, the inconsistencies between studies again underscore the need for future research with hormonal verification. Morever, adequate sample sizes, and standardized exercise protocols will improve both consistency and help develop and promote the inclusion of women in vascular research.

## 1. Introduction

It is well established that premenopausal women—those still experiencing regular menstrual cycles—have a lower incidence of cardiovascular disease compared to age-matched men, a difference largely attributed to the protective effects of estrogen on cardiovascular health [1], particularly through its influence on endothelial function—an important early indicator of cardiovascular risk [2].

Regular physical activity improves endothelial function and reduces cardiovascular risk [2]. However, sex-based differences in vascular adaptations to exercise remain poorly understood. Male and female sex hormones appear to differentially regulate endothelial and vascular smooth muscle function, potentially resulting in distinct exercise-induced vascular responses [3,4,5,6]. 

The acute effects of exercise on endothelial function are less well understood and appear to be influenced by factors such as age, health status, fitness level, and exercise parameters, including the intensity, duration, and volume of exercise [7]. Understanding these vascular responses can help predict individual adaptations and reveal vascular plasticity [8].

Given estrogen’s key role in vascular regulation [3,5], most studies involving women test participants during the early menstrual phase (days 1–7) to reduce hormonal variability [9,10]. However, premenopausal women experience cyclical fluctuations in estrogen, with both high and low estrogen phases throughout the menstrual cycle [11]. As such, limiting research to the early phase may restrict the inclusion of women and overlook important physiological responses. This narrative review examines vascular responses (changes on vascular function) to acute exercise across menstrual phases to promote greater inclusivity and precision in future research. 

## 2. Estrogen and Its Impact on the Endothelium

The endothelium, a monolayer of cells lining blood vessels, plays a crucial role in regulating vascular homeostasis by releasing vasoactive substances, most notably nitric oxide (NO). NO is a key vasodilator that maintains vascular tone, supports endothelial integrity, and promotes cardiovascular health. Increased NO bioavailability enhances vasodilation, protects against endothelial dysfunction, and lowers cardiovascular risk [2]. 

Estrogen, particularly 17β-estradiol, promotes NO synthesis by stimulating endothelial nitric oxide synthase (eNOS) expression via estrogen receptor alpha (ERα), or by inducing eNOS phosphorylation through PI3K/Akt signaling [12]. Moreover, it reduces oxidative stress [1] and enhances the function of endothelial progenitor cells (EPCs), contributing to endothelial cell proliferation and facilitating vascular repair [13]. 

These effects contribute to sex differences in vascular function, fluctuating with hormonal shifts throughout the menstrual cycle [14] and changing with age [15]. Premenopausal women generally exhibit superior endothelial function compared to age-matched men [16] likely due to estrogen’s vascular protective properties [1]. Indeed, flow-mediated dilation (FMD), a non-invasive assessment of NO-dependent endothelial function [17], is generally higher in premenopausal women compared to age-matched men [16,18], while some studies also suggest an increase from the early (low estrogen) to late follicular (high estrogen) phase, aligning with peak estrogen levels [18,19,20,21]. A more detailed discussion of these phases will follow in the subsequent section.

The onset of menopause (the final menstrual period) reduces estrogen levels, leading to lower NO bioavailability and increasing oxidative stress [1,15]. Estrogen loss also elevates sympathetic activity and α-adrenergic vasoconstriction, raising blood pressure both at rest and during exercise in postmenopausal women (normally defined as ≥12 months without menstruation) [22], affecting arterial compliance. Moreover, postmenopausal women often exhibit reduced arterial compliance and endothelial function [16,23,24,25,26] compared to pre-menopausal women. Endothelial function tends to decline shortly after menopause, due to reduced estrogen levels [27], and this decline may continue throughout the menopausal transition. While lower levels are often reported in late menopause, the most pronounced drop appears to occur between the pre-menopause and early menopause stages [26].

Estrogen therapy in postmenopausal women appears to mitigate some of the above adverse effects [28], helping improve FMD and lower oxidative stress [27], particularly when combined with exercise training [29,30]. However, the cardiovascular effects of estrogen therapy remain contentious, as delayed initiation may elevate cardiovascular risk, whereas early intervention appears protective [28,31]. Notably, the route of administration, type, dosage, and combination with progestins all significantly influence the therapy’s outcomes [32]. 

## 3. Menstrual Phases Overview and the Physiological Effect of the Phases on Endothelial Function

**Figure 1:**
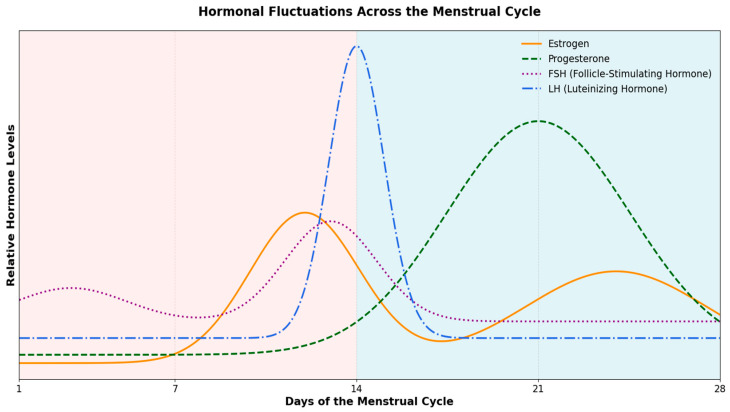
Hormonal Fluctuations Across the Menstrual Cycle: Estrogen, Progesterone, Follicle-Stimulating Hormone (FSH), and (Luteinizing Hormone) LH Dynamics. Throughout the menstrual cycle, FSH gradually increases during the follicular phase, reaching a peak just before ovulation, and then declines. LH remains relatively low until a dramatic surge occurs around the time of ovulation. Estrogen rises progressively during the follicular phase, peaking just prior to ovulation, before dipping and experiencing a secondary rise during the luteal phase. Progesterone remains low in the follicular phase, then increases sharply after ovulation, peaking in the luteal phase, and ultimately declines.

A typical 28-day menstrual cycle consists of three phases: (a) the follicular phase (~days 1–13), (b) ovulation (~day 14), and (c) the luteal phase (~days 15–28), each characterized by distinct hormonal fluctuations [11]. Briefly, during the early follicular phase, estrogen and progesterone levels are initially low (~days 1–7), with estrogen gradually rising as ovulation nears (~days 8–13), while progesterone remains low. Around ovulation (~day 14), estrogen drops slightly while levels of luteinizing hormone (LH) and follicle-stimulating hormone (FSH) increase significantly [33]. In the luteal phase (~days 4–6 post-LH peak; ~days 15–22 of a typical menstrual cycle), progesterone rises sharply after ovulation, while estrogen shows a slight decrease but remains relatively high. In the later part of the luteal phase (~days 8–13 post-LH peak; ~days 23–28 of a typical menstrual cycle), progesterone declines, and estrogen levels rise again. These hormonal fluctuations throughout the menstrual cycle are illustrated in Figure 1. It is, however, crucial to acknowledge that despite this “text-book” description, there exists considerable inter- and intra-individual variability in menstrual cycle length and hormonal profiles.

Hormonal fluctuations can impact vascular function [34]. Estrogen rises from the early to the late follicular phase (~days 7–14), improving endothelial function, as shown by enhanced FMD or elevated NO levels [18,19,20,21,35,36]. During ovulation, endothelial integrity is maintained despite a slight estrogen drop [37]. In the early luteal phase, reduced estrogen may impair vascular function, evidenced by decreased FMD and arterial distensibility [21,37]. As progesterone rises, it may counteract estrogen’s effects, with one study even showing similar FMD during the mid-luteal phase (days 23–25) and early follicular phase [38]. However, vascular function may improve in the mid- to late luteal phase [21,39,40]. Overall, the above studies suggest that vascular function may be modulated by estrogen levels, with potential antagonism from progesterone.

Discrepancies exist, with some studies showing no phase effects on the vasculature [32,41,42,43]. Moreover, a meta-analysis [34] reports a slight increase in endothelial function from early to late follicular phases, with no significant microvascular changes during the luteal phase. These discrepancies may reflect cycle-to-cycle variability [44], complicating the identification of phase-dependent vascular effects.

## 4. Vascular Responses to Acute Exercise

Regular exercise benefits endothelial health [2], but the acute effects of a single session can vary, showing improvement, impairment, or no change, depending on exercise type, intensity, participant health, and timing of assessment [7]. Briefly, a single bout of moderate-intensity exercise generally increases endothelial function, while higher intensity exercise may induce oxidative stress resulting in transient decrease in endothelial function before returning to baseline levels, particularly in individuals with cardiovascular risk factors compared to young healthy individuals. More details about how these parameters affect acute vascular responses have been analyzed in the following resources [7,45,46]. 

Although most of the studies assessing endothelial function in response to acute exercise are conducted in men, some studies showed superior vascular responses following acute bout of exercise in premenopausal women compared to age-matched men [47,48,49]. Furthermore, moderate-intensity exercise reduced circulating CD62E+ microparticles (an endothelial damage marker) in women but not in men [50]. Additionally, Doonan et al. [51] reported a higher arterial stiffness (as measured by pulse wave velocity; PWV) in men compared to premenopausal women. However, some studies report distinct findings, such as Hwang et al. [52] who observed impaired brachial artery FMD in women post-exercise, and Shenouda et al. [53] who found no significant changes in brachial artery FMD in either sex. 

While there is evidence of baseline differences in vascular responses between postmenopausal women and both premenopausal women and age-matched men, as well as differences in vascular adaptations to exercise training [3,5,6], data on vascular responses to acute exercise remain limited. For example, Yoo et al. [54] found that brachial artery FMD was reduced after acute exercise in men but remained unchanged in postmenopausal women, suggesting a potential protective effect despite menopause. Earlier studies within women report that although FMD declines from pre- to postmenopausal status at baseline, acute moderate-intensity exercise can still enhance FMD—often to a greater extent in postmenopausal than premenopausal women [55,56]. In contrast, Serviente et al. [57] reported that perimenopausal women showed enhanced brachial artery FMD and reduced inflammatory markers post-exercise, while postmenopausal women showed no FMD improvement and increased platelet-derived microparticles, suggesting estrogen decline may impair these responses [6,27].

A major confounder in studying vascular responses is menstrual cycle phase, which influences endothelial function through hormonal fluctuations [34] and thereby affects acute exercise responses. However, many of the studies mentioned above either fail to report cycle phase [47,52] do not control for it [50], or assess women in only the follicular phase [48,49,51,53]. Such methodological inconsistencies limit the generalizability of findings, increasing variability in sex-specific research outcomes. This highlights a critical need for enhanced standards of practice in women’s health research [58].

The following section reviews vascular responses to acute exercise across menstrual cycle phases in premenopausal women.

## 5. Acute Exercise in Females and Menstrual Phases

This section shifts the focus to premenopausal women, examining how hormonal fluctuations during different menstrual phases impact vascular responses to acute exercise. While we recognize the value of a systematic review approach, we opted for a narrative review due to the limited number of relevant studies and considerable heterogeneity in study designs, exercise protocols, participant characteristics, and vascular assessment methods. This section includes studies examining vascular responses to acute exercise in premenopausal females across different menstrual cycle phases, using protocols such as handgrip, leg extension, resistance exercise, and cycling. The studies summarized below and in Table 1 examine how hormonal fluctuations influence exercise-induced increases in blood flow and vasodilation [2], along with other measures such as arterial stiffness and vascular conductance. Table 1 also notes whether participants had cardiovascular risk factors (e.g., obesity), which can affect vascular responses.

Although FMD is commonly used to assess vascular endothelium responses, as it reflects NO-mediated vasodilation [17], we have identified only a limited number of studies that have utilized this measure for acute exercise across the menstrual cycle. Two notable studies, by D’Urzo et al. [41] and Khaksar et al. [59], examined whether estrogen fluctuations affect FMD during distinct follicular sub-phases. Specifically, D’Urzo et al. [41] assessed brachial artery FMD during the early (days 2–7) and late follicular phases (days 13–14), while Khaksar et al. [59] employed a similar timeline, comparing days (1–5) with days (10–14). Despite hormonal divergence, both studies reported no significant differences in FMD, suggesting that estrogen alone may have limited influence on endothelial function in response to acute exercise. 

In addition to FMD, Weggen et al. [60] examined vascular responses (blood flow and arterial diameter changes), using passive leg movement and handgrip exercise in young healthy women during the early (days 1–7) and late (days 12–14) follicular phases. The authors also measured vascular conductance, accounting for mean arterial pressure. Both protocols showed no significant menstrual cycle effects on blood flow, dilation, or vascular conductance, consistent with earlier findings. Similarly, Limberg et al. [40] found no menstrual phase differences in forearm blood flow and vascular conductance during steady-state dynamic exercise. Furthermore, Shiozawa et al. [61] observed no phase-dependent variations in vascular responses assessed via celiac artery blood flow and conductance during moderate-intensity knee-extension exercise across the follicular (days 1–4) and luteal (days 18–22) phases in young healthy women. Gonzales et al. [62] investigated forearm blood flow and vascular conductance across the follicular (days 7–14) and luteal (days 18–24) phases using dynamic handgrip exercise, paired either with a placebo or with L-citrulline supplementation (a non-essential amino acid that serves as a substrate for eNOS) [63]. In the study, L-citrulline supplementation raised plasma arginine levels, suggesting potential vascular benefits, but did not significantly alter forearm blood flow across menstrual phases or improve vascular responses compared to placebo. Together, these findings indicate that menstrual cycle phase has minimal impact on vascular responses to low- to moderate-intensity exercise.

Restaino et al. [64] compared forearm blood flow and forearm vascular conductance (FVC) in obese women and healthy controls. During early menstruation (~days 1–5), obese women showed lower blood flow and conductance at rest and during isotonic handgrip exercise to exhaustion. However, these vascular responses improved in the proliferative phase (1 week after the early menstruation phase: typically, ~5–12 days, no direct hormonal measurements were conducted), suggesting rising estrogen levels have a protective effect. The findings imply obesity may impair vascular responses to acute exercise [7] while higher estrogen levels in the early menstruation phase may help moderate these effects [3,5].

Given the limited data in FMD and/or other measures such as exercise-induced blood flow, which are heavily related to endothelium and NO bioavailability [17,41], we also reviewed studies using alternative vascular measures such as PWV, a reliable measurement of arterial stiffness [65]. These measurements are strongly influenced by neural regulation, making them sensitive to blood pressure changes.

Okamoto et al. [66] found that high-intensity resistance training increased PWV 30 and 60 min post-exercise during the follicular phase (days 1–5) but not in the luteal phase (days 20–24) in healthy premenopausal women. This suggests that higher estrogen levels in the luteal phase may reduce exercise-induced blood pressure spikes, possibly through enhanced NO production [5,6] and by counteracting sympathetic nervous system (SNS) activation during resistance exercise. Unlike low-to-moderate aerobic exercise, resistance training may cause sharp, immediate blood pressure spikes [67] partly due to vessel compression, the Valsalva maneuver, and a strong exercise pressor reflex [68,69]. These surges can overactivate the SNS, raising PWV, but higher estrogen levels are linked to reduced SNS activity, potentially lessening these effects [70].

Park et al. [71] assessed central vascular conductance using thoracic impedance cardiography during cycling at 60% VO_2_max in the early follicular (days 2–4) and late ovulation (days 10–13) phases. They found total vascular conductance decreased in the early follicular phase but improved during ovulation. Importantly, unlike the above studies that assessed vascular conductance locally via blood flow, this study measured systemic conductance using cardiac output, which limits interpretation of changes in exercised muscles and local vasodilation. Hormone levels were not directly measured; however, these differences may be explained by estrogen’s influence on autonomic nervous system regulation, where lower estrogen during early follicular phase promotes sympathetic nervous system activation and peripheral vasoconstriction.

In summary, vascular responses assessed by endothelium-dependent measures such as FMD or shear-rate dilation (e.g., passive leg or handgrip) appears to remain stable across the menstrual cycle during moderate-intensity exercise. However, vascular responses measured by methods more reliant on neural regulation and arterial pressure, such as PWV or vascular conductance, particularly during high-intensity exercise and resistance training, which are likely associated with increased sympathetic activity, may be modulated by estrogen. This protective effect likely reflects estrogen’s influence on autonomic function and vascular tone.

**Table 1:** Summary of Studies Investigating Menstrual Phase Effects on Vascular Responses to Exercise.

Study	Participants’	Menstrual Phase and Assessment	Exercise Protocol	Outcome Measures	Measurement Timing	Key Findings
D’Urzo et al., 2018 [41]	Healthy premenopausal women (n = 12)	Early Follicular (Days 2–7); Late Follicular (Days 13–14); Hormonal levels via blood samples	Handgrip MVC for 6 min (1 s on/5 s off)	FMD	Baseline, each minute for 6 min	No phase differences in vascular responses.
Restaino et al., 2022 [64]	Healthy (n = 20), and obese (n = 9) premenopausal women	Early menstrual phase (~Days 1–5); Proliferative Phase (~Days 7–12); Hormonal levels via blood samples	Handgrip: 2 minwarm-up, increase (0.25 W/min) until failure	FVC	Final 15 s of exercise	Reduced blood flow in obese women during early menses; improved in proliferative phase
Okamoto et al., 2017 [66]	Healthy premenopausal women (n = 9)	Follicular (Days 1–5); Luteal (Days 20–24); Hormonal levels via blood samples	Warm up, bench press (80% 1RM, 5 × 5), biceps curl (70% 1RM, 5 × 10)	PWV	Baseline, 30 min, 60 min post-exercise	Increase PWV at 30- and 60 minpost-exercise in follicular phase only.
Gonzales et al., 2020 [62]	Healthy premenopausal women (n = 24)	Follicular (Days 7–14); Luteal (Days 18–24); Hormonal levels via blood samples	Handgrip (10% MVC, 5 min); Citrulline supplementation (6 g/day for 7 days) vs. placebo	FVC, FBF, Plasma Arginine	Baseline, last 30s of exercise	No phase differences or citrulline effects on vascular measures.
Weggen et al., 2023 [60]	Healthy premenopausal women (total n = 21; n = 11 measured in two phases)	Early Follicular (Days 1–7); Late Follicular (Days 12–14); Hormonal levels via blood samples	Passive leg movement (PLM); handgrip (3 kg, 6 kg, 3 min each)	Vascular conductance, blood flow (femoral and brachial)	Baseline, during (PLM) or last minute each stage (handgrip)	No phase differences in vascular responses.
Park et al., 2017 [71]	Healthy premenopausal women (n = 10)	Early Follicular (Days 1–4); Late Follicular (Days 10–13) Calendar-based tracking	2 minwarm-up; 30 min cycling 60% VO_2_peak	Total vascular conductance	5, 10, 15 min post-exercise	Higher total vascular conductance in late follicular phase.
Shiozawa et al., 2023 [61]	Healthy premenopausal women (n = 11)	Early Follicular (Days 1–4); Mid-Luteal (Days 18–22); Menstrual phase via Ovulation Predictor Kit	Dynamic leg exercise at 30% HRR, 4 min	Celiac artery blood flow and vascular conductance	Baseline, every 1 min during exercise	No phase differences in vascular responses.
Limberg et al., 2010 [40]	Healthy premenopausal women (n = 9)	Early Follicular (Day 3 ± 0.3); Early Luteal (Day 15 ± 0.8); Hormonal levels via blood samples	Handgrip 15% and 30% MVC 7 min; Phenylephrine or clonidine infusion during final 3 min	FBF, FVC	Baseline, continuously during exercise, prior and during infusions	No phase difference at baseline and during exercise prior infusions. Vasoconstrictor response to clonidine was lower in the early luteal phase compared to the early follicular phase at 15% MVC.

MVC, maximum voluntary contraction; FMD, flow-mediated dilation; 1RM, 1 repetition maximum; FVC, forearm vascular conductance; FBF, forearm blood flow; PLM, passive leg movement; VO_2_peak, peak volume oxygen uptake; HRR, heart rate reserve.

## 6. Potential Mechanisms

Although estrogen levels fluctuate across the menstrual cycle, acute moderate-intensity exercise does not significantly alter endothelial function between phases. In contrast, central hemodynamic measures like PWV and vascular conductance are more sensitive to these changes, showing stronger responses when estrogen is high. During exercise, vascular tone reflects a balance between vasodilation—partly via increased NO bioavailability—and vasoconstriction from sympathetic activation. Estrogen supports this balance by enhancing NO availability [12] and reducing sympathetic activity [70].

In terms of autonomic regulation and sex-based differences, premenopausal women generally experience lower levels of muscle sympathetic nerve system (SNS)-induced vasoconstriction compared to men [72], a disparity often attributed to higher estrogen levels. However, this protective effect seems to diminish following menopause, resulting in vascular responses that more closely align with those observed in men [22,72,73,74]. Within the menstrual cycle, elevated estrogen levels during the luteal phase have been associated with reduced sympathetic neural activity [70]. Notably, Chidambaram et al. [75] demonstrated that despite an increase in circulating renin-angiotensin system components, vasoconstrictive responses at the tissue level may be attenuated during the luteal phase (days 15–24) compared to the follicular phase (days 3–6), which could explain the vasodilatory effect observed in some studies in the luteal phase. 

Exercise modality and intensity also influence acute endothelial responses. High-intensity exercise and resistance training stimulate the SNS and increase endothelin-1 levels (a vasoconstrictive agent), which can override vasodilatory mechanisms and impair vascular responses [76]. However, during phases of elevated estrogen, the hormone may counteract these adverse effects, as observed in Okamoto et al. [66]. In contrast, low- to moderate-intensity aerobic exercise typically adopts a more balanced autonomic response, promoting vasodilation without relying heavily on estrogen for regulation [40]. However, current methodological limitations challenge the interpretation of study findings. Notably, the common practice of estimating menstrual cycle phases rather than employing direct hormonal profiling introduces significant validity concerns, underscoring the critical need for more rigorous research methodologies [77]. Overall, current evidence on acute exercise-induced vascular responses across the menstrual cycle is limited and primarily focused on dynamic exercise, particularly handgrip, which restricts our ability to assess the effects of different exercise modalities. As exercise type and intensity may differentially influence vascular responses [7], future research should aim to standardize protocols, modalities, and intensities while incorporating phase-specific considerations.

Moreover, inter-individual variability in menstrual cycle length and hormonal fluctuations further complicates the interpretation of vascular responses to exercise. The duration and hormonal profile of menstrual cycles can vary significantly, particularly in estradiol levels, which exert an influence on endothelial function. Indeed, Liu et al. [44] observed that intra-individual variability in estradiol levels across cycles contributes to inconsistent endothelial responses, making it quite challenging to draw definitive conclusions based on a single cycle.

## 7. Conclusions

The current body of research reveals important but nuanced relationships between menstrual cycle phase and vascular responses to exercise. Although the number of studies is limited and methodologies vary, current evidence, particularly regarding shear-induced responses such as FMD, generally suggests stable vascular outcomes across the menstrual cycle. This may indicate that strict control of menstrual phase is not always necessary in certain contexts, which could help improve the generalizability of exercise studies in women. However, further high-quality research is needed to confirm these observations. In contrast, measures with stronger neural mediation, specifically vascular conductance and PWV, generally demonstrate greater estrogen sensitivity. Phases with higher estrogen levels are linked to improved blood pressure and arterial stiffness; therefore, standardizing cycle timing or including measured hormone levels as covariates may enhance accuracy and interpretation. Nonetheless, significant inconsistencies exist across investigations, likely due to small sample sizes, lack of hormonal verification phase, and varied exercise protocols, limit firm conclusions. Additionally, substantial inter-individual variability in menstrual cycle length and hormonal fluctuations, further complicates phase-specific interpretations. To advance the field, future studies should use hormonal verification of menstrual phases, ensure adequate sample sizes, standardize exercise protocols, and adopt longitudinal designs to capture within-subject variability across multiple cycles.

## Abbreviations

The following abbreviations are used in this manuscript:

1RM	One Repetition Maximum
EPCs	Endothelial Progenitor Cells
ERα	Estrogen Receptor Alpha
eNOS	Endothelial Nitric Oxide Synthase
FBF	Forearm Blood Flow
FMD	Flow-Mediated Dilation
FSH	Follicle-Stimulating Hormone
FVC	Forearm Vascular Conductance
HRR	Heart Rate Reserve
LH	Luteinizing Hormone
MVC	Maximum Voluntary Contraction
NO	Nitric Oxide
PI3K/Akt	Phosphoinositide 3-Kinase/Protein Kinase B
PLM	Passive Leg Movement
PWV	Pulse Wave Velocity
SNS	Sympathetic Nervous System
VO_2_peak	Peak Volume of Oxygen Uptake
